# Supplementary material for: The Genetic Architecture of a Congenital Heart Defect Is Related to Its Fitness Cost
Source: Genes (Basel). 2021 Aug 31;12(9):1368. doi: 10.3390/genes12091368 (PMC8467714; doi:10.3390/genes12091368)
Supplement: Supplementary file 1 [file genes-12-01368-s001.zip › genes-1335808-Supplement.pdf]

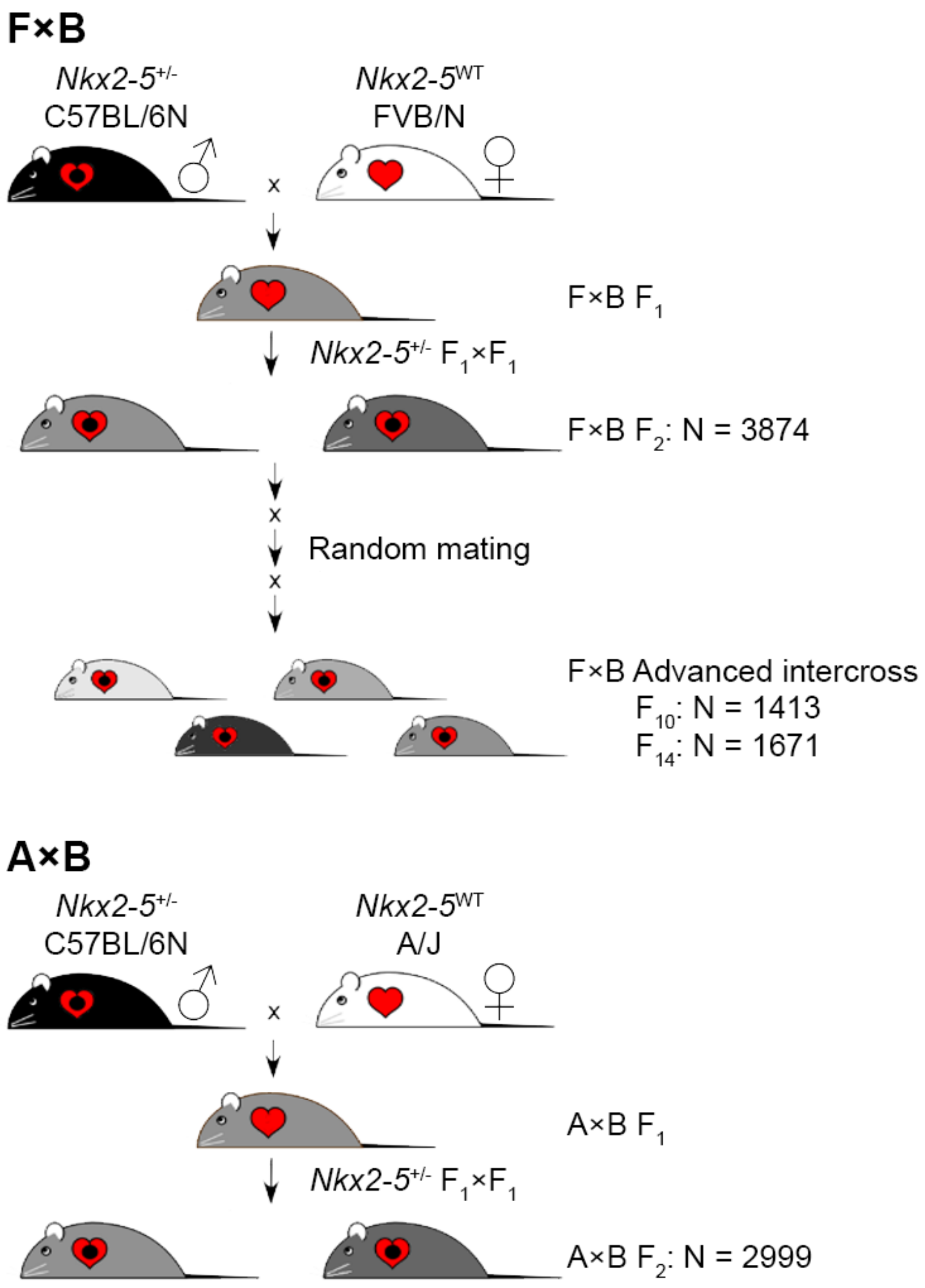

Supplementary Figure S1. Breeding scheme for inbred strain intercrosses. We produced F_1 hybrids by crossing wild-type FVB/N (F) or A/J (A) females to *Nkx2-5*$^{+/-}$ C57BL/6N (B) males. We phenotyped newborn *Nkx2-5*$^{+/-}$ F_2 pups from both A×B and F×B intercrosses and F_{10} and F_{14} pups from the F×B advanced intercross. The advanced intercross was produced by random mating of non-siblings and non-first cousins beginning in the F_3 and F_4 generations. The number of *Nkx2-5*$^{+/-}$ pups phenotyped in each generation is shown.

a

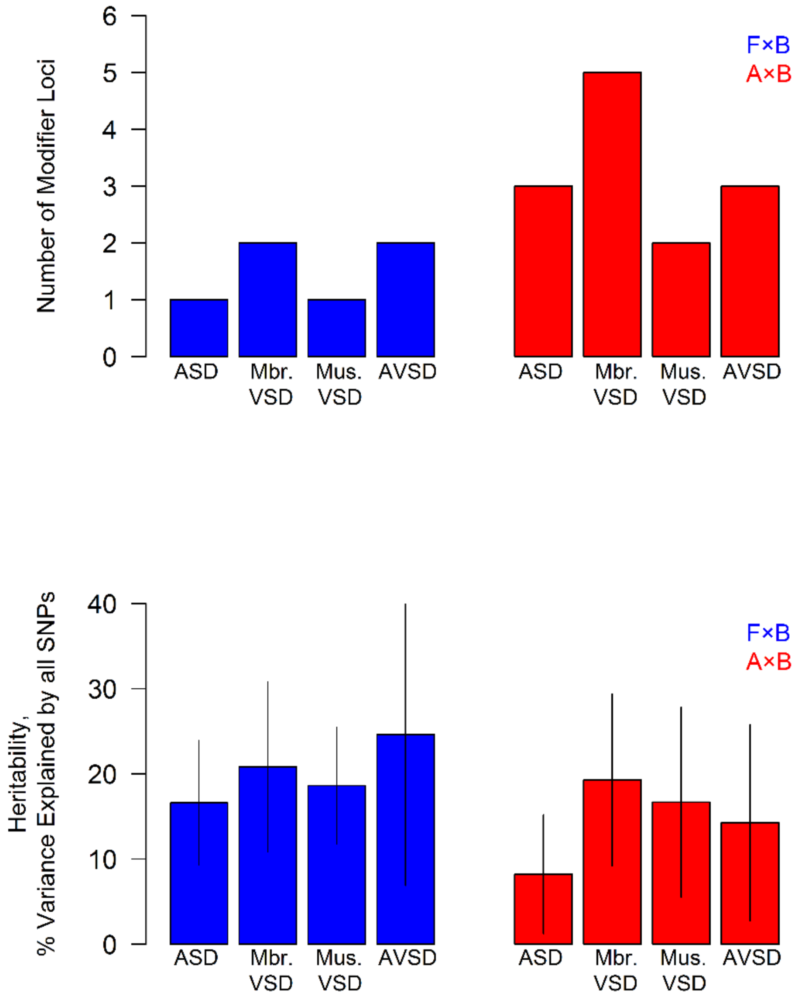

b

Supplementary Figure S2. Neither **a,** the number of significant and suggestive loci detected nor **b,** the heritability explained by all genotyped SNPs varies with the severity of a defect. Error bars are 95% C.I.

Supplementary Figure S3. The observed incidences at two-locus genotypes of ASD, membranous and muscular VSD, and AVSD are shown between every pair of suggestive or genome-wide significant modifier loci found in both crosses. In the two cases where there is only one suggestive locus, the locus is plotted against the next most significant locus in the cross.

ASD: F×B

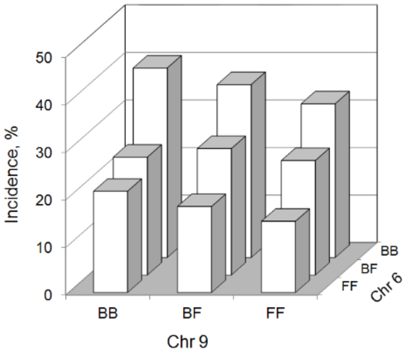

ASD: A×B

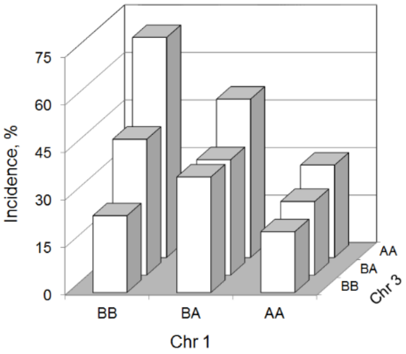

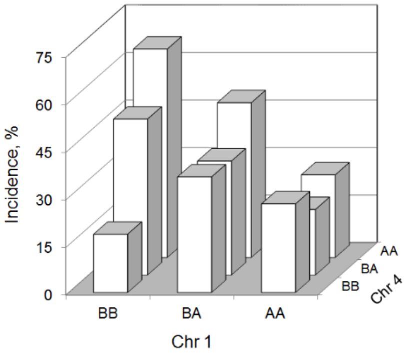

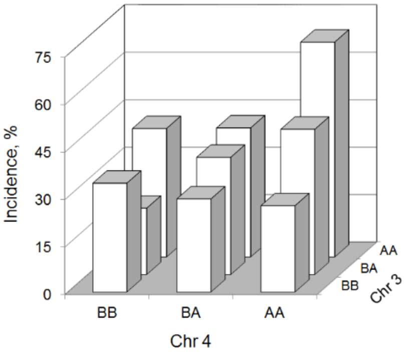

Membranous VSD: F×B

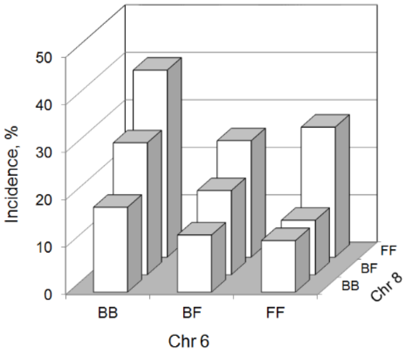

Supplementary Figure S3 **Akhirome *et al***

Membranous VSD: A×B

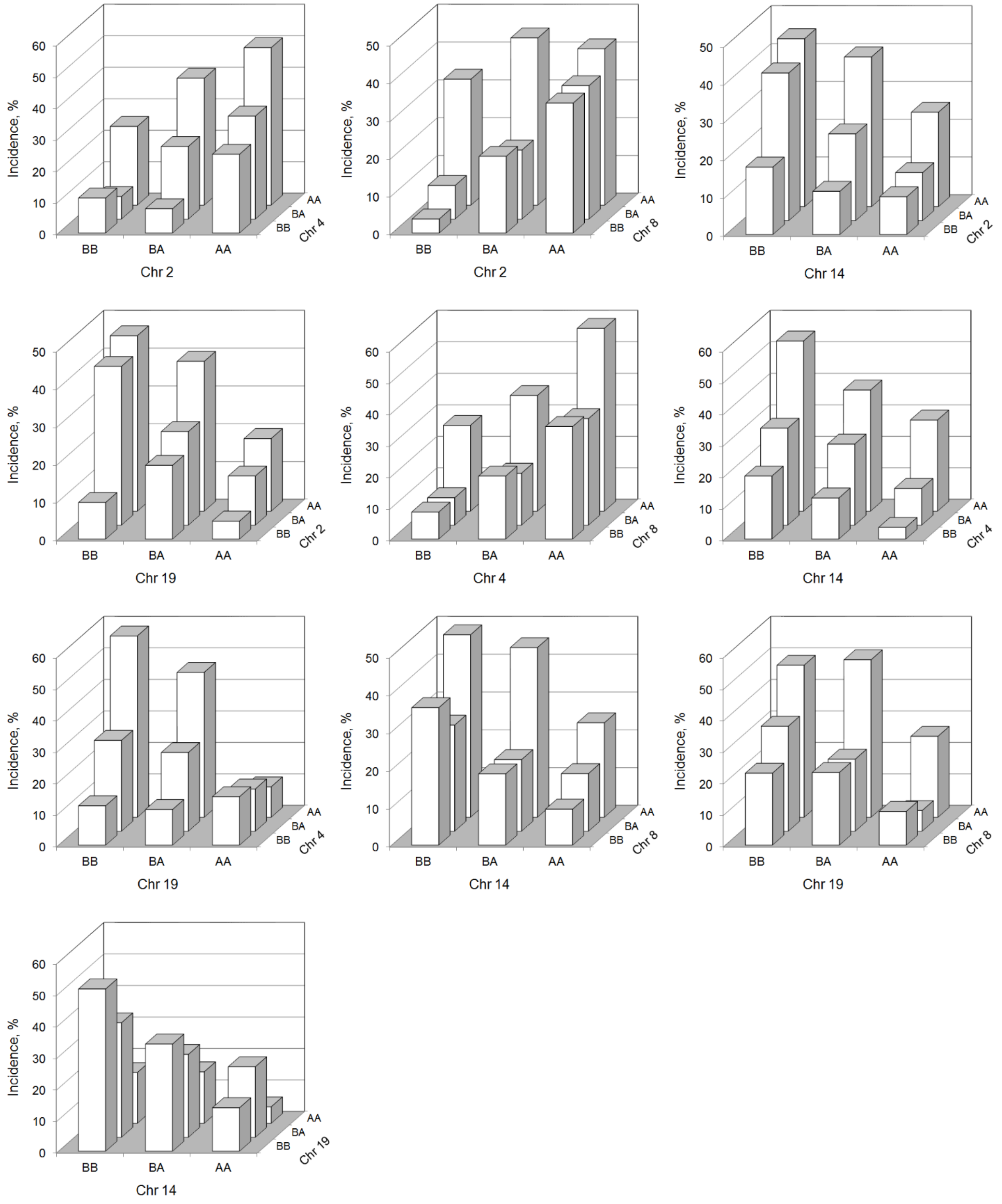

Muscular VSD: F×B

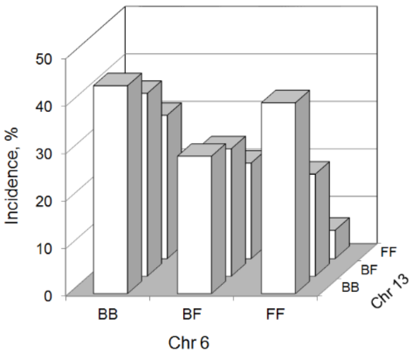

Muscular VSD: A×B

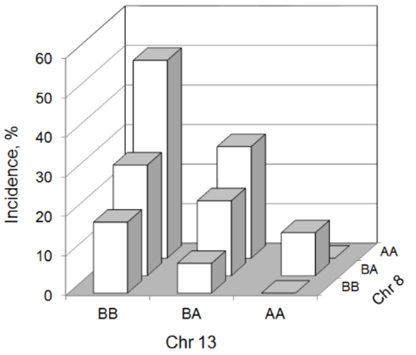

AVSD: F×B

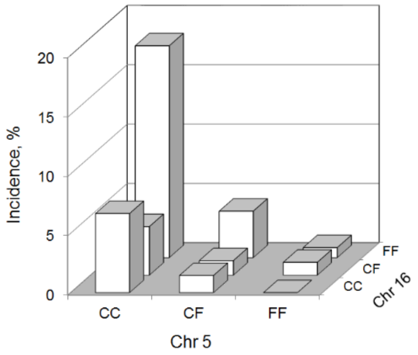

AVSD: A×B

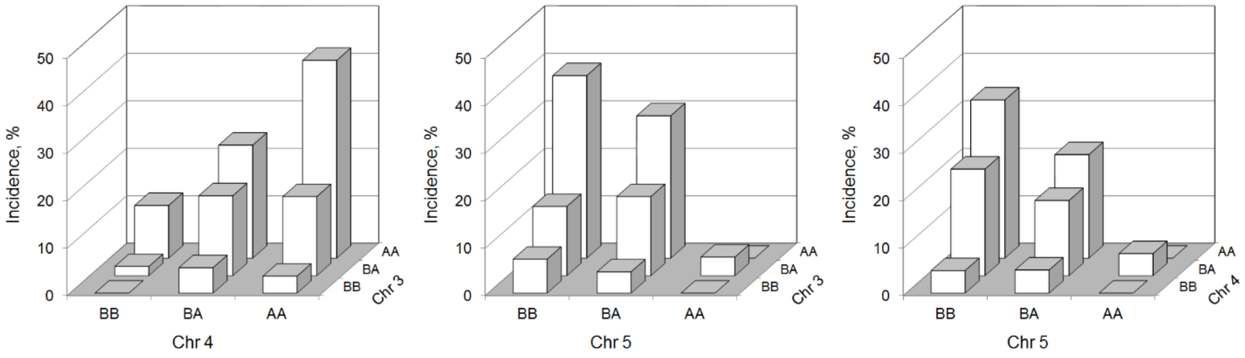

Table S1
SNPs in the A×B F2 intercross

SNP, rsID	Chromosome	Centimorgans, Cox sex-averaged	Minor allele frequency
rs13475818	1	16.447	0.474
rs3163007	1	32.307	0.465
rs13475934	1	39.511	0.456
rs3684360	1	54.7	0.473
rs3701630	1	69.107	0.470
rs6238909	1	84.931	0.469
rs13476354	2	10.3282	0.475
rs13459062	2	21.806	0.475
rs3659784	2	62.098	0.490
rs27349879	2	75.407	0.497
rs3659658	2	89.467	0.484
rs3676540	3	6.8289	0.467
rs29807239	3	14.268	0.472
rs3716258	3	24.097	0.473
rs3706378	3	33.755	0.481
rs13477291	3	43.5124	0.479
rs31135898	3	53.0931	0.500
rs30509660	3	63.036	0.495
rs3685116	3	72.3035	0.483
rs13477517	3	81.6622	0.490
rs27682879	4	7.5557	0.485
rs27807782	4	14	0.471
rs3719378	4	45.706	0.485
rs6195490	4	57.6654	0.491
rs27610338	4	69.051	0.485
rs13478067	4	86.167	0.482
rs13478133	5	9.968	0.463
rs13478182	5	18.159	0.467
rs13478245	5	26.822	0.475
rs3684754	5	36.0449	0.467
rs13478337	5	41.8192	0.475
rs3655541	5	51.434	0.488
rs3715788	5	60.5724	0.497
rs6237258	5	68.258	0.494
rs13478585	5	85.2381	0.499
rs6172481	6	1.806	0.495
rs6271324	6	15.478	0.491
rs13478830	6	32.556	0.487
rs6292642	6	48.928	0.486
rs6361894	6	63.435	0.481
rs13479152	7	12.4855	0.499

rs32082888	7	25.4805	0.493
rs3675728	7	39.8674	0.496
rs4137968	7	54.459	0.499
rs13479530	7	73.187	0.482
rs3023176	8	7.587	0.464
rs13479701	8	22.3824	0.456
rs13479794	8	31.55	0.458
rs13479955	8	50.074	0.475
rs13479979	8	56.197	0.483
rs13480121	9	16.3358	0.500
rs30458137	9	32.7528	0.498
rs33720457	9	40.876	0.485
rs3710939	9	57.983	0.492
rs13480488	10	3.006	0.472
rs13459187	10	5.143	0.473
rs29329268	10	5.8055	0.469
rs13480521	10	7.8605	0.474
rs13459119	10	9.754	0.479
rs29325765	10	47.05	0.465
rs13480773	10	62.2764	0.460
rs26864121	11	13.3116	0.476
rs13480991	11	25.492	0.484
rs13481091	11	43.7211	0.483
rs27059631	11	58.9	0.500
rs3706128	11	70.2894	0.494
rs13481256	11	82.957	0.489
rs3678500	12	9.9405	0.471
rs13481388	12	15.533	0.471
rs13481459	12	22.8396	0.473
rs6333493	12	28.726	0.476
rs13481595	12	45.422	0.478
rs3705279	13	7.2572	0.493
rs13459140	13	17.8688	0.454
rs13481783	13	21.21	0.493
rs29237897	13	35.7893	0.486
rs29229165	13	50.4979	0.494
rs13482037	13	67.21	0.493
rs30637674	14	7.08	0.485
rs13482119	14	18.657	0.481
rs3715961	14	23.9629	0.484
rs3653455	14	34.614	0.482
rs6306220	14	44.0387	0.470
rs13482356	14	56.165	0.478
rs32275246	15	8.538	0.483
rs13482486	15	9.493	0.483
rs13482587	15	21.146	0.486
rs32164372	15	38.015	0.488

rs3695692	15	48.0369	0.500
rs32677126	16	9.636	0.489
rs4169065	16	21.7827	0.486
rs4189683	16	34.357	0.496
rs4210345	16	45.7803	0.492
rs13459152	17	22.593	0.474
rs13483049	17	29.7243	0.466
rs33633158	17	45.4911	0.463
rs6358426	18	10.1	0.486
rs29976462	18	23.8762	0.488
rs3656892	18	42.5727	0.488
rs30997811	19	18.3527	0.472
rs30415214	19	35.9722	0.463
rs30931570	19	48.4583	0.469

Table S1
SNPs in the F×B F2 and advanced intercrosses

SNP rsID	Chromosome	Centimorgans, Cox sex-averaged	Minor allele frequency
rs6269442	1	1.614	0.405
rs13475701	1	1.744	0.405
rs13475706	1	1.889	0.411
rs3684358	1	2.06	0.412
rs13475729	1	3.749	0.450
rs3671256	1	5.009	0.431
rs13475750	1	6.153	0.429
rs6404446	1	6.442	0.430
rs31949292	1	8.486	0.446
rs6173215	1	8.692	0.443
rs31917015	1	10.425	0.429
rs6166266	1	10.731	0.417
rs3677683	1	10.932	0.413
rs6384194	1	11.319	0.408
rs3695988	1	11.345	0.408
rs4137502	1	11.461	0.410
rs31837951	1	12.249	0.432
rs3707642	1	12.426	0.436
rs3681732	1	12.953	0.430
rs3683997	1	14.842	0.414
rs4222295	1	17.369	0.417
rs13475823	1	18.581	0.426
rs13475827	1	20.081	0.425
rs13475834	1	20.661	0.428
rs13475847	1	23.851	0.450
rs13475851	1	25.709	0.449
rs31383619	1	25.876	0.448
rs3724092	1	25.91	0.448
rs13475866	1	26.334	0.453
rs6322485	1	32.063	0.467
rs3163007	1	32.307	0.469
rs6288543	1	34.187	0.479
rs6312657	1	34.68	0.493
rs13475931	1	39.196	0.490
rs13475934	1	39.511	0.486
rs13475936	1	39.78	0.482
rs13475939	1	40.572	0.481
rs13475946	1	41.565	0.482
rs13475952	1	42.11	0.496
rs3723062	1	43.848	0.415
rs32600524	1	44.067	0.416
rs6195073	1	44.7	0.459

rs31435980	1	44.9793	0.459
rs13475980	1	46.189	0.458
rs6268443	1	47.188	0.497
rs3694065	1	48.814	0.496
rs30596698	1	49.306	0.496
rs31680156	1	49.601	0.494
rs3685919	1	50.674	0.497
rs13476061	1	50.919	0.490
rs3674655	1	52.113	0.458
rs3678634	1	52.718	0.461
rs4137908	1	54.799	0.463
rs13464873	1	55.969	0.478
rs3699561	1	56.934	0.484
rs3724826	1	57	0.484
rs13476119	1	58.159	0.471
rs8250053	1	58.391	0.471
rs30968449	1	60.722	0.477
rs13476141	1	61.841	0.482
rs6382880	1	62.409	0.483
rs13476152	1	62.56	0.483
rs3672697	1	63.104	0.462
rs30986578	1	63.3199	0.462
rs6411476	1	63.404	0.462
rs13476184	1	65.303	0.485
rs31544365	1	67.667	0.485
rs31299650	1	69.032	0.480
rs13476229	1	76.767	0.437
rs3707910	1	79.926	0.491
rs3669108	1	81.044	0.485
rs13476265	1	83.595	0.440
rs13476273	1	84.91	0.429
rs3667164	1	93.199	0.444
rs13476300	1	96.082	0.380
rs6180557	1	97.9052	0.405
rs27096879	2	2.2384	0.499
rs6213083	2	2.316	0.499
rs4136817	2	2.515	0.477
rs6359983	2	3.79	0.495
rs6240512	2	8.426	0.491
rs3674936	2	14.52	0.405
rs13459062	2	21.806	0.402
rs32868446	2	22.274	0.399
rs13476442	2	22.742	0.408
rs13476439	2	24.323	0.439
rs13476454	2	24.77	0.462
rs13476467	2	26.974	0.435
rs13476472	2	27.478	0.418

rs6265423	2	27.693	0.418
rs27901975	2	29.025	0.465
rs3725341	2	29.897	0.425
rs13476503	2	30.511	0.426
rs27929044	2	30.694	0.426
rs3718711	2	31.734	0.426
rs3672719	2	33.018	0.455
rs33395966	2	36.722	0.470
rs6222797	2	38.403	0.467
rs4136610	2	41.073	0.464
rs3664661	2	42.714	0.470
rs13476580	2	44.165	0.493
rs3722345	2	48.304	0.491
rs3667007	2	49.029	0.491
rs8273639	2	49.444	0.496
rs13476621	2	49.887	0.496
rs6404809	2	50.023	0.496
rs13476636	2	50.636	0.460
rs13476639	2	51.239	0.466
rs13476649	2	52.255	0.464
rs6378047	2	53.194	0.498
rs27359225	2	53.374	0.498
rs13476666	2	53.746	0.498
rs13476689	2	56.063	0.487
rs13476691	2	56.306	0.485
rs13476698	2	56.748	0.476
rs33283452	2	56.948	0.476
rs4138562	2	57.704	0.478
rs3699172	2	58.312	0.479
rs3149106	2	59.399	0.485
rs8279354	2	59.823	0.487
rs3658927	2	60.369	0.500
rs6228179	2	60.677	0.483
rs6401493	2	61.384	0.497
rs13476762	2	62.081	0.457
rs3661596	2	63.204	0.389
rs3665528	2	63.278	0.389
rs6363071	2	71.292	0.459
rs6209325	2	73.369	0.468
rs27349879	2	75.407	0.439
rs13476860	2	77.376	0.408
rs3695266	2	78.55	0.346
rs13476874	2	80.116	0.321
rs3664408	2	81.738	0.296
rs13476889	2	85.007	0.325
rs27311433	2	88.761	0.361
rs3726974	2	89.685	0.375

rs13476928	2	97.782	0.450
rs13476932	2	98.14	0.448
rs3679483	2	102.647	0.403
rs27681559	2	102.9288	0.406
rs13476963	3	2.104	0.396
rs13476969	3	2.406	0.393
rs13476973	3	2.935	0.383
rs6248752	3	2.967	0.383
rs13476985	3	3.23	0.379
rs3659988	3	4.094	0.402
rs13476997	3	4.99	0.390
rs6235984	3	7.393	0.435
rs13477030	3	10.99	0.449
rs3660588	3	13.426	0.462
rs29807239	3	14.268	0.442
rs13477043	3	15.29	0.418
rs29919622	3	16.101	0.420
rs4223883	3	16.434	0.420
rs6246699	3	17.452	0.432
rs6324747	3	19.211	0.480
rs30263314	3	20.388	0.487
rs13477097	3	21.275	0.487
rs30057783	3	21.726	0.492
rs4139913	3	21.749	0.491
rs6241331	3	23.549	0.496
rs4223969	3	26.26	0.497
rs13477126	3	27.287	0.496
rs13477138	3	29.092	0.448
rs13477143	3	29.302	0.449
rs13477154	3	30.058	0.450
rs6212539	3	30.168	0.480
rs31430997	3	30.245	0.481
rs13477167	3	30.735	0.485
rs6198234	3	32.419	0.433
rs6264454	3	32.571	0.433
rs13477201	3	33.735	0.427
rs3706378	3	33.755	0.427
rs13477210	3	34.419	0.415
rs13477217	3	34.881	0.429
rs13477224	3	35.847	0.444
rs13477233	3	37.284	0.445
rs13477251	3	38.955	0.424
rs13477268	3	40.211	0.403
rs3022964	3	42.089	0.416
rs3726226	3	42.961	0.418
rs4138887	3	45.048	0.449
rs13477299	3	45.247	0.461

rs3701653	3	45.461	0.474
rs29690864	3	47.761	0.465
rs13477321	3	47.987	0.464
rs3676545	3	48.528	0.455
rs3698700	3	49.118	0.460
rs13477357	3	50.381	0.450
rs3708412	3	50.969	0.429
rs30157172	3	52.425	0.431
rs13477404	3	60.271	0.500
rs13477421	3	62.12	0.485
rs30509660	3	63.036	0.485
rs3676039	3	63.124	0.485
rs13477439	3	64.048	0.480
rs3090379	3	66.233	0.488
rs6290401	3	66.587	0.495
rs3657112	3	72.448	0.423
rs13477494	3	76.859	0.431
rs13477506	3	79.346	0.392
rs30801216	3	81.07	0.394
rs13477517	3	81.6622	0.443
rs30353245	3	82.066	0.477
rs3660863	4	3.374	0.434
rs6287606	4	7.098	0.435
rs27682879	4	7.532	0.464
rs27682826	4	7.534	0.464
rs13477596	4	8.599	0.469
rs13477602	4	9.709	0.478
rs27748877	4	10.325	0.482
rs13477617	4	11.276	0.490
rs3701432	4	12.487	0.493
rs27801920	4	13.275	0.475
rs3663744	4	13.517	0.475
rs27807782	4	14	0.447
rs3674908	4	14.005	0.447
rs13477643	4	17.003	0.458
rs3698283	4	23.004	0.452
rs27856136	4	24.49	0.457
rs3677770	4	29.364	0.466
rs3712541	4	32.814	0.461
rs27935605	4	33.958	0.480
rs6254381	4	34.002	0.481
rs13477838	4	42.52	0.406
rs13477866	4	45.65	0.428
rs28076769	4	45.6667	0.428
rs13477883	4	47.779	0.402
rs6324470	4	49.475	0.422
rs6226080	4	50.643	0.404

rs3710617	4	51.378	0.429
rs6381371	4	53.085	0.453
rs3692563	4	54.658	0.495
rs3675629	4	56.727	0.465
rs6195490	4	57.6654	0.497
rs13477959	4	57.707	0.498
rs6355837	4	63.346	0.473
rs32281313	4	66.216	0.477
rs3663950	4	67.994	0.465
rs13478002	4	68.924	0.461
rs27610338	4	69.051	0.455
rs4224864	4	70.21	0.409
rs3719891	4	75.668	0.461
rs3023025	4	77.015	0.471
rs13478089	4	84.321	0.456
rs13478067	4	85.611	0.470
rs13478068	4	86.105	0.475
rs6279100	4	88.605	0.452
rs3664617	5	2.537	0.412
rs6402980	5	2.883	0.397
rs6223482	5	3.199	0.396
rs3714258	5	3.933	0.393
rs3687916	5	6.151	0.402
rs13478139	5	10.162	0.475
rs13478154	5	13.069	0.483
rs13469943	5	16.96	0.425
rs3700706	5	17.391	0.428
rs13459085	5	17.941	0.405
rs13478182	5	18.159	0.406
rs6256504	5	21.165	0.414
rs13478205	5	22.295	0.424
rs6215373	5	24.09	0.426
rs13478223	5	25.023	0.427
rs3659933	5	25.74	0.427
rs29521905	5	25.98	0.430
rs3714001	5	26.89	0.445
rs3664008	5	29.397	0.451
rs259632697	5	30.567	0.448
rs29584438	5	30.975	0.447
rs3090667	5	31.824	0.442
rs6187409	5	32.229	0.437
rs33138184	5	32.845	0.448
rs3707918	5	38.702	0.488
rs3153758	5	40.4907	0.469
rs29678187	5	40.926	0.464
rs3721607	5	42.897	0.426
rs29560457	5	44.025	0.440

rs13478384	5	44.297	0.438
rs3673049	5	44.318	0.438
rs13478388	5	44.712	0.438
rs6232866	5	45.474	0.455
rs13478402	5	47.492	0.454
rs29502845	5	47.559	0.455
rs3661241	5	47.74	0.478
rs3705458	5	48.326	0.499
rs13478416	5	48.461	0.499
rs13478451	5	53.197	0.497
rs13478463	5	54.517	0.498
rs13478473	5	55.949	0.497
rs6362200	5	55.994	0.495
rs13478479	5	56.285	0.479
rs6255362	5	60.0892	0.490
rs32146173	5	60.445	0.487
rs13478518	5	66.363	0.454
rs13478540	5	74.362	0.470
rs13478546	5	76.004	0.468
rs4225536	5	77.062	0.473
rs6298689	5	78.785	0.461
rs3656197	5	80.281	0.468
rs13478567	5	81.309	0.468
rs13478573	5	82.5642	0.467
rs13478583	5	85.1753	0.463
rs3718776	5	89.193	0.452
rs3661828	6	1.448	0.442
rs13478602	6	1.65	0.442
rs6172481	6	1.888	0.449
rs3699833	6	2.513	0.445
rs13478617	6	3.436	0.444
rs13478631	6	4.899	0.440
rs13478641	6	7.199	0.468
rs3655269	6	8.073	0.431
rs13478656	6	8.808	0.421
rs3671709	6	11.736	0.411
rs13478677	6	11.989	0.410
rs13478684	6	12.318	0.402
rs30945099	6	15.021	0.368
rs13478705	6	15.022	0.368
rs13478709	6	15.265	0.380
rs3704635	6	15.43	0.388
rs6330932	6	16.438	0.441
rs13478719	6	17.777	0.476
rs6238771	6	21.815	0.428
rs13478732	6	21.865	0.428
rs13478745	6	23.816	0.429

rs13478753	6	24.558	0.470
rs30910255	6	27.764	0.448
rs13478819	6	32.381	0.413
rs3672029	6	32.53	0.413
rs13478830	6	32.556	0.412
rs3699367	6	33.047	0.411
rs6377140	6	35.012	0.418
rs6181382	6	35.526	0.411
rs31002289	6	35.969	0.410
rs13459097	6	36.95	0.325
rs30605552	6	37.538	0.318
rs13478882	6	39.97	0.350
rs13478891	6	40.922	0.352
rs13478893	6	41.108	0.355
rs6239023	6	42.752	0.375
rs6349084	6	44.804	0.389
rs13478919	6	45.115	0.384
rs4138572	6	45.6	0.383
rs6292642	6	48.559	0.366
rs6208251	6	48.778	0.364
rs13478971	6	51.615	0.366
rs13478977	6	52.302	0.335
rs6393943	6	53.019	0.320
rs6401637	6	53.749	0.315
rs13478997	6	55.551	0.309
rs13479014	6	59.24	0.389
rs6389420	6	61.973	0.354
rs3681620	6	62.834	0.382
rs30316697	6	63.358	0.387
rs3722480	6	63.612	0.388
rs6339546	6	64.542	0.358
rs13479053	6	64.794	0.376
rs30015657	6	65.8784	0.391
rs3023102	6	66.215	0.396
rs13479071	6	68.53	0.403
rs3672808	6	69.744	0.447
rs6329892	6	74.038	0.470
rs3658783	6	76.543	0.483
rs30160101	6	77.5675	0.468
rs30848564	6	77.701	0.461
rs6265387	6	78.094	0.442
rs30950360	6	78.865	0.452
rs3700068	7	2.536	0.469
rs3714915	7	2.617	0.469
rs13479105	7	2.689	0.469
rs6384973	7	3.058	0.468
rs3658362	7	7.76	0.465

rs3689218	7	9.441	0.491
rs13479153	7	13.06	0.480
rs31465998	7	15.8664	0.466
rs32390275	7	16.524	0.462
rs6239372	7	20.611	0.438
rs32082888	7	25.4805	0.447
rs13479208	7	27.892	0.444
rs3719256	7	28.86	0.468
rs31893514	7	31.277	0.472
rs3699938	7	32.593	0.445
rs13479243	7	32.8436	0.446
rs13479251	7	32.929	0.446
rs3663313	7	33.441	0.414
rs31944466	7	33.556	0.417
rs3714908	7	34.645	0.446
rs13479319	7	38.358	0.463
rs3676254	7	41.543	0.408
rs13479339	7	43.462	0.442
rs13479347	7	43.881	0.449
rs13479355	7	44.319	0.449
rs13479358	7	45.126	0.456
rs13479363	7	45.693	0.469
rs6213614	7	47.085	0.491
rs13479385	7	48.729	0.475
rs13479395	7	50.443	0.482
rs32517623	7	50.648	0.481
rs4226783	7	51.622	0.490
rs13479414	7	52.15	0.467
rs3707067	7	53.897	0.405
rs13479427	7	54.309	0.421
rs4137968	7	54.459	0.414
rs3713052	7	54.621	0.406
rs6357312	7	54.711	0.407
rs13479461	7	58.644	0.437
rs6275579	7	67.508	0.495
rs13479506	7	68.039	0.492
rs3682038	7	69.108	0.498
rs3716088	7	76.319	0.484
rs13479540	7	77.8094	0.456
rs31680975	7	81.137	0.388
rs3663988	7	84.321	0.314
rs31198744	7	88.854	0.392
rs6153168	8	3.092	0.456
rs3023176	8	7.587	0.489
rs13479619	8	7.914	0.485
rs3657963	8	8.446	0.437
rs13479625	8	9.266	0.423

rs33566717	8	10.583	0.438
rs4140004	8	11.326	0.438
rs3661760	8	11.48	0.438
rs13479656	8	14.032	0.415
rs13479673	8	16.533	0.414
rs4227096	8	18.218	0.402
rs3706948	8	18.826	0.402
rs13479701	8	22.3824	0.418
rs3665028	8	22.606	0.419
rs3667738	8	23.504	0.406
rs13479716	8	23.71	0.406
rs3703811	8	23.87	0.406
rs3666140	8	24.316	0.406
rs6386110	8	24.757	0.439
rs3719401	8	27.887	0.452
rs13479755	8	28.863	0.455
rs31276910	8	29.03	0.455
rs13479768	8	29.246	0.455
rs3707439	8	30.761	0.430
rs3672639	8	30.936	0.441
rs13479793	8	31.514	0.467
rs13479794	8	31.55	0.467
rs13479811	8	33.219	0.480
rs3656875	8	33.567	0.492
rs3699406	8	34.067	0.494
rs3667475	8	34.488	0.479
rs13479830	8	35.127	0.443
rs36926924	8	37.45	0.429
rs13479871	8	41.475	0.473
rs6257357	8	41.611	0.473
rs13479882	8	42.129	0.467
rs13479884	8	42.33	0.469
rs6391152	8	43.853	0.458
rs13479922	8	44.877	0.454
rs4137596	8	46.663	0.427
rs6285803	8	48.12	0.436
rs6287320	8	48.493	0.435
rs13479947	8	49.503	0.443
rs3706149	8	50.061	0.443
rs13479955	8	50.074	0.443
rs3669235	8	50.253	0.443
rs13479956	8	50.34	0.443
rs3662808	8	56.991	0.439
rs6237645	8	57.389	0.444
rs33401493	8	63.315	0.481
rs3708073	8	70.716	0.444
rs6310696	8	70.824	0.446

rs6377872	8	71	0.450
rs3705725	8	72.309	0.458
rs31833030	8	72.3393	0.458
rs13480087	9	7.064	0.452
rs13480092	9	7.305	0.452
rs13480095	9	7.4084	0.456
rs8270115	9	7.927	0.475
rs13480103	9	9.511	0.473
rs3088801	9	10.106	0.473
rs6404775	9	14.314	0.499
rs6385855	9	15.145	0.487
rs3655898	9	18.165	0.418
rs30437276	9	20.114	0.445
rs6413270	9	20.806	0.426
rs33646953	9	24.4263	0.443
rs13462199	9	24.739	0.444
rs13480173	9	25.485	0.451
rs6395817	9	25.803	0.464
rs13480179	9	26.352	0.464
rs13480186	9	27.114	0.496
rs3723670	9	28.839	0.496
rs13480208	9	29.932	0.470
rs13480217	9	31.394	0.496
rs6224703	9	32.333	0.498
rs30458137	9	32.7528	0.496
rs3714012	9	33.18	0.493
rs3655717	9	35.34	0.493
rs6174757	9	36.954	0.492
rs6355445	9	39.122	0.482
rs3721056	9	39.7	0.488
rs13480271	9	40.24	0.496
rs33720457	9	40.876	0.489
rs3724833	9	41.977	0.479
rs13480285	9	42.583	0.483
rs13480298	9	43.863	0.484
rs3676124	9	45.286	0.485
rs3695050	9	46.021	0.460
rs3700596	9	46.512	0.494
rs3669564	9	47.114	0.496
rs13480340	9	47.798	0.470
rs13480345	9	48.379	0.471
rs13480351	9	48.893	0.461
rs29789189	9	50.284	0.420
rs3689336	9	50.613	0.423
rs13480365	9	51.376	0.423
rs3717654	9	54.162	0.413
rs30343415	9	55.0782	0.431

rs13480399	9	57.487	0.475
rs13480407	9	59.514	0.461
rs13480428	9	63.934	0.449
rs6320810	9	67.636	0.402
rs3669563	9	69.841	0.447
rs13459114	9	72.67	0.487
rs6304156	9	73.664	0.484
rs6185923	10	0.438	0.471
rs29368538	10	1.298	0.464
rs3721803	10	1.696	0.464
rs13480488	10	3.006	0.442
rs3664101	10	3.154	0.439
rs4228112	10	4.417	0.446
rs13459187	10	5.143	0.443
rs29329268	10	5.8055	0.441
rs3699409	10	5.979	0.440
rs13480521	10	7.8605	0.466
rs13459119	10	9.293	0.487
rs13480534	10	9.7958	0.489
rs3679120	10	10.4	0.494
rs13480547	10	11.755	0.494
rs13480554	10	12.941	0.498
rs13480566	10	16.478	0.472
rs13480578	10	18.922	0.465
rs13480579	10	19.237	0.427
rs13480601	10	23.19	0.463
rs13480605	10	23.8239	0.470
rs3696307	10	27.314	0.492
rs13480621	10	30.574	0.460
rs13480627	10	34.7364	0.408
rs13480630	10	35.028	0.403
rs29332616	10	37.031	0.446
rs13480652	10	38.38	0.449
rs13480657	10	38.917	0.473
rs3717445	10	40.766	0.477
rs13480678	10	41.5	0.478
rs3679902	10	41.674	0.478
rs3089366	10	45.073	0.481
rs13480702	10	45.499	0.477
rs29325765	10	47.05	0.464
rs13480710	10	47.545	0.459
rs13480712	10	48.536	0.459
rs13480716	10	48.973	0.460
rs13480722	10	50.35	0.476
rs3710293	10	51.414	0.442
rs3688351	10	54.723	0.431
rs6290359	10	56.295	0.443

rs6243755	10	56.692	0.443
rs29353606	10	61.545	0.359
rs3680872	10	61.556	0.359
rs29360855	10	67.701	0.360
rs3697243	10	73.832	0.376
rs3676330	10	76.442	0.383
rs13480836	11	2.495	0.424
rs13480837	11	2.735	0.424
rs13480863	11	5.918	0.424
rs26925478	11	6.126	0.425
rs3023249	11	6.662	0.424
rs13480875	11	7.335	0.431
rs29389113	11	10.513	0.470
rs13480910	11	14.219	0.411
rs3700830	11	17.794	0.450
rs13459123	11	18.321	0.468
rs26822994	11	18.8679	0.468
rs3723833	11	19.079	0.467
rs13480968	11	22.232	0.469
rs6359329	11	23.638	0.491
rs13480996	11	25.809	0.499
rs3654344	11	25.922	0.500
rs26898753	11	27.389	0.500
rs13481009	11	27.986	0.500
rs13481011	11	28.08	0.499
rs13481033	11	32.472	0.452
rs4228731	11	33.04	0.451
rs3684076	11	33.7	0.468
rs3697686	11	36.25	0.456
rs3711357	11	37.947	0.475
rs13481076	11	40.59	0.404
rs6197743	11	42.96	0.413
rs13481109	11	46.074	0.392
rs13481119	11	46.826	0.365
rs13481145	11	52.843	0.332
rs3714299	11	58.424	0.443
rs27059631	11	58.9	0.444
rs3661058	11	62.745	0.440
rs6393948	11	67.403	0.477
rs3698446	11	68.645	0.477
rs6386362	11	70.093	0.482
rs6370458	11	71.91	0.497
rs13481227	11	73.596	0.416
rs3672597	11	75.82	0.391
rs3699056	11	79.467	0.375
rs13481256	11	82.957	0.443
rs3712881	11	84.892	0.476

rs13481303	12	5.626	0.436
rs3706330	12	7.88	0.457
rs13481321	12	7.923	0.458
rs13481324	12	8.117	0.460
rs3717860	12	8.495	0.456
rs13481363	12	10.543	0.436
rs3089800	12	11.63	0.425
rs13481371	12	12.768	0.425
rs6223000	12	14.927	0.433
rs13481388	12	15.533	0.434
rs6311081	12	16.814	0.435
rs3658100	12	17.537	0.435
rs13481406	12	18.11	0.451
rs13481408	12	18.369	0.451
rs29148707	12	20.993	0.485
rs3670749	12	22.242	0.464
rs13481459	12	22.8396	0.462
rs3706319	12	25.397	0.452
rs29158995	12	25.941	0.464
rs13481496	12	26.705	0.479
rs13481514	12	28.892	0.460
rs13481527	12	30.34	0.472
rs3655558	12	32.585	0.447
rs13481541	12	33.626	0.455
rs3662628	12	35.986	0.492
rs29176418	12	36.457	0.495
rs13481565	12	38.807	0.491
rs29184538	12	43.833	0.437
rs3670410	12	44.326	0.436
rs13481588	12	45.007	0.435
rs13481592	12	45.198	0.439
rs6207869	12	45.478	0.444
rs13481599	12	46.455	0.448
rs13481604	12	49.82	0.483
rs3716084	12	50.269	0.462
rs13481614	12	51.183	0.486
rs13481632	12	56.892	0.461
rs6390948	12	59.558	0.469
rs13481655	12	61.147	0.493
rs3023711	12	62.619	0.480
rs29249127	13	1.733	0.465
rs6215262	13	2.1	0.464
rs13481668	13	2.487	0.464
rs13481673	13	2.837	0.442
rs6348604	13	5.492	0.475
rs3023379	13	6.3652	0.468
rs13481715	13	6.898	0.464

rs6314295	13	7.214	0.458
rs3705279	13	7.2572	0.458
rs3679784	13	7.543	0.460
rs3663223	13	9.387	0.437
rs13481767	13	16.31	0.485
rs13481783	13	21.21	0.487
rs3712907	13	21.327	0.498
rs3688207	13	21.883	0.492
rs6411274	13	24.301	0.493
rs6244558	13	24.703	0.492
rs3693942	13	29.453	0.490
rs3690198	13	32.531	0.499
rs4229817	13	34.532	0.474
rs29730990	13	34.544	0.474
rs13481871	13	37.601	0.460
rs13481880	13	40.224	0.489
rs13481883	13	40.871	0.488
rs13481908	13	43.36	0.472
rs29247893	13	43.678	0.472
rs29565666	13	44.743	0.472
rs29932904	13	45.235	0.471
rs6288319	13	46.504	0.449
rs6316213	13	47.483	0.431
rs13481943	13	47.76	0.431
rs29229165	13	50.4979	0.404
rs13481961	13	50.925	0.399
rs13481968	13	52.195	0.404
rs13481983	13	55.812	0.425
rs13459786	13	58.109	0.424
rs13482005	13	59.736	0.403
rs13482022	13	64.043	0.432
rs6247696	13	64.656	0.433
rs13482028	13	64.842	0.434
rs13482035	13	66.772	0.433
rs13482037	13	67.21	0.437
rs4230144	14	4.71	0.477
rs6322899	14	5.762	0.488
rs6290836	14	6.421	0.480
rs30637674	14	7.08	0.479
rs3687889	14	11.062	0.481
rs30721414	14	11.9357	0.459
rs13482096	14	12.574	0.442
rs3692121	14	14.111	0.447
rs13482104	14	15.674	0.470
rs6396829	14	16.608	0.466
rs13482119	14	18.657	0.473
rs13482143	14	22.258	0.475

rs13482159	14	23.452	0.480
rs3715961	14	23.9629	0.474
rs13482170	14	25.173	0.458
rs3666583	14	26.733	0.470
rs13482191	14	27.71	0.470
rs13482194	14	28.17	0.469
rs13482216	14	33.165	0.495
rs3672425	14	35.55	0.472
rs13482247	14	37.396	0.445
rs6284633	14	37.5422	0.445
rs13459144	14	40.316	0.443
rs13482265	14	42.277	0.424
rs13482284	14	43.806	0.420
rs3708535	14	44.007	0.421
rs13482296	14	44.438	0.421
rs6395984	14	45.189	0.410
rs6291434	14	45.459	0.410
rs13482311	14	45.801	0.422
rs30835762	14	45.957	0.424
rs13482327	14	47.839	0.442
rs3692362	14	49.712	0.441
rs30942806	14	52.61	0.441
rs13482354	14	56.151	0.460
rs3654132	14	56.158	0.460
rs13482375	14	57.817	0.475
rs6256423	14	58.78	0.429
rs6169105	14	60.717	0.419
rs3665550	14	62.625	0.417
rs13482398	14	63.475	0.438
rs13482407	14	65.852	0.418
rs31594308	15	1.843	0.477
rs13482418	15	1.849	0.477
rs3665826	15	2.032	0.474
rs31671106	15	3.823	0.500
rs31927778	15	4.44	0.492
rs13482431	15	5.575	0.446
rs3711814	15	6.248	0.410
rs31608298	15	7.706	0.424
rs32187076	15	7.773	0.424
rs13482455	15	7.869	0.424
rs3715857	15	8.139	0.424
rs13482477	15	8.543	0.424
rs32275246	15	8.617	0.424
rs13482497	15	11.032	0.414
rs13482504	15	12.282	0.427
rs32084485	15	12.5423	0.430
rs6188239	15	14.103	0.444

rs13482529	15	15.274	0.438
rs32312277	15	16.751	0.459
rs13482543	15	16.809	0.459
rs13482549	15	17.395	0.442
rs3707900	15	19.919	0.436
rs13482585	15	21.175	0.466
rs13482595	15	25.03	0.460
rs31824103	15	29.0311	0.398
rs13482628	15	29.421	0.391
rs13482637	15	32.182	0.393
rs13482661	15	37.583	0.425
rs3665030	15	37.745	0.427
rs32164372	15	38.015	0.431
rs6276391	15	38.245	0.434
rs13482695	15	43.934	0.451
rs13482704	15	45.108	0.461
rs13482712	15	46.484	0.487
rs6285067	15	49.907	0.443
rs13482751	15	58.021	0.471
rs4152638	16	2.426	0.386
rs4152790	16	2.475	0.386
rs4231060	16	2.889	0.390
rs4157412	16	3.4148	0.389
rs4158907	16	3.509	0.389
rs32677126	16	9.636	0.414
rs4165081	16	12.309	0.409
rs4165279	16	12.645	0.407
rs4167031	16	19.714	0.461
rs4168640	16	21.374	0.481
rs4170308	16	22.749	0.476
rs4171440	16	24.104	0.489
rs4177651	16	27.831	0.477
rs4179075	16	28.385	0.474
rs4182243	16	30.376	0.453
rs4183448	16	30.604	0.453
rs4185639	16	31.431	0.469
rs4186744	16	32.099	0.468
rs3696661	16	32.767	0.458
rs6163640	16	32.971	0.453
rs4189277	16	34.227	0.460
rs4189683	16	34.357	0.456
rs4191367	16	34.819	0.440
rs4193069	16	36.067	0.427
rs4194417	16	36.445	0.427
rs4197416	16	37.958	0.417
rs4197715	16	38.865	0.410
rs4200124	16	40.292	0.418

rs4201998	16	40.906	0.418
rs6317052	16	45.623	0.373
rs4211364	16	45.892	0.388
rs4211664	16	46.759	0.417
rs3680665	16	46.916	0.418
rs4212102	16	47.413	0.413
rs4213268	16	49.112	0.397
rs4214396	16	49.568	0.397
rs4217260	16	51.637	0.371
rs4219905	16	54.059	0.387
rs3712360	16	54.488	0.387
rs3696981	16	57.427	0.397
rs4136382	17	2.001	0.428
rs13482845	17	2.515	0.465
rs33373629	17	3.756	0.468
rs3672987	17	17.806	0.386
rs6390174	17	19.311	0.397
rs3702604	17	19.559	0.388
rs33178797	17	19.602	0.388
rs6409750	17	22.923	0.449
rs13483016	17	25.749	0.433
rs6272475	17	27.907	0.433
rs3714226	17	28.823	0.420
rs3727008	17	38.866	0.483
rs3675634	17	42.179	0.495
rs3691628	17	44.65	0.459
rs13483103	17	45.436	0.477
rs13483117	17	49.683	0.490
rs13483119	17	50.304	0.491
rs13483131	17	51.965	0.491
rs13483135	17	53.085	0.461
rs13483144	17	56.362	0.478
rs4231720	17	56.873	0.468
rs33117354	17	59.733	0.460
rs6288047	17	60.459	0.457
rs6397044	17	60.64	0.446
rs13483183	18	2.302	0.414
rs29628302	18	3.578	0.406
rs4231742	18	5.653	0.411
rs13483212	18	6.068	0.406
rs30271401	18	8.002	0.412
rs6358426	18	10.034	0.442
rs13483244	18	11.555	0.468
rs4138020	18	11.996	0.446
rs3722973	18	17.694	0.487
rs3664831	18	19.514	0.497
rs6220251	18	21.395	0.453

rs3676196	18	22.966	0.413
rs4231834	18	23.625	0.458
rs29976462	18	23.8762	0.451
rs30176009	18	24.277	0.441
rs13483364	18	28.334	0.379
rs29626928	18	34.209	0.429
rs8237402	18	35.096	0.428
rs3688789	18	37.483	0.428
rs3691542	18	39.503	0.475
rs13483432	18	45.629	0.456
rs13483436	18	48.825	0.434
rs13459193	18	50.842	0.473
rs13483446	18	51.811	0.476
rs3720876	18	53.237	0.474
rs13483466	18	55.727	0.440
rs29667966	18	57.837	0.499
rs13483482	18	58.209	0.495
rs30456650	19	3.3776	0.487
rs6236348	19	4.441	0.487
rs30517739	19	7.103	0.466
rs6316813	19	8.177	0.434
rs30697936	19	8.771	0.490
rs31280015	19	10.1503	0.483
rs3686467	19	12.273	0.475
rs30604569	19	13.2036	0.474
rs13483556	19	13.705	0.474
rs6309315	19	18.426	0.425
rs6293693	19	20.923	0.394
rs3090325	19	21.258	0.416
rs30746021	19	21.7875	0.414
rs13459194	19	26.625	0.384
rs6237466	19	27.057	0.385
rs30396271	19	29.792	0.397
rs30364728	19	29.987	0.398
rs30920120	19	32.228	0.395
rs3655407	19	34.251	0.404
rs30415214	19	35.9722	0.400
rs3656289	19	36.17	0.399
rs3687275	19	36.617	0.392
rs13461374	19	36.6975	0.390
rs13483643	19	38.427	0.356
rs3023496	19	41.195	0.369
rs6304326	19	47.937	0.456
rs30931570	19	48.4583	0.453
rs13483682	19	51.073	0.435
rs249259485	19	56.384	0.454

Table S2. Modifier loci by cross and defect.
A×B, LOD. F×B, -log(p).
Bold, genome-wide significant. Non-bold, suggestive.
*, estimated marker. The closest SNP is indicated.
The locus interval is bounded by the 1 LOD or -log(p) drop from the peak SNP.

Cross	Defect	Peak SNP	Locus interval	LOD or -log(p)	Risk Allele	Candidate genes
A×B	ASD	rs6238909	chr1:125665904-182054904	2.88	B	*Csrp1, Lefty1, Lefty2, Pitx2, Vangl2*
A×B	ASD	rs31135898	chr3:52939690-158052844	3.22	A	*Bmpr1b, Notch2, Pifo, Pitx2*
A×B	ASD	rs13478067*	chr4:124165816-154991070	2.69	A	
A×B	Mbr.VSD	rs3659784	chr2:31172022-152809607	**3.42**	A	*Actc1, Acvr1, Bbs5, Jag1, Lrp2, Mkks, Ttc21b, Zeb2*
A×B	Mbr.VSD	rs27610338	chr4:100332046-154991070	**5.60**	A	*Ptch2*
A×B	Mbr.VSD	rs13479701	chr8:14759093-62208384	**3.81**	A	*Hand2, Vegfc,*
A×B	Mbr.VSD	rs13482119	chr14:17628076-67124319	2.92	B	*Bmp4, Bmpr1a, Esco2, Gata4, Ift88, Myh6*
A×B	Mbr.VSD	rs30415214	chr19:23376527-53761523	**3.55**	B	*Acta2, Fgf8*
A×B	Mus.VSD	rs13479794	chr8:14759093-108947107	**3.77**	A	*Bbs2, Gdf1, Hand2, Nfatc3, Sall1, Vegfc*
A×B	Mus.VSD	rs13482037	chr13:96099181-118789220	**5.87**	B	*Fgf10, Isl1*
A×B	AVSD	rs13477291	chr3:75914491-136196844	**4.98**	A	*Notch2, Pifo, Pitx2*
A×B	AVSD	rs27610338*	chr4:100332046-154991070	**3.40**	A	*Ptch2*
A×B	AVSD	rs13478337	chr5:35506765-129965381	**4.08**	B	*Cc2d2a, Evc, Evc2. Pkd2, Ptpn11, Med13l, Tbx3, Tbx5*
F×B	ASD	rs6304156	chr9:121915911-123153990	3.02	B	
F×B	Mbr.VSD	rs6292642	chr6:94055997-111299794	**6.12**	B	*Foxp1*
F×B	Mbr.VSD	rs3657963	chr8:15279371-17579118	**4.98**	F	*Csmd1*
F×B	Mus.VSD	rs6349084	chr6:85961013-104553235	3.13	B	*Prickle2, Foxp1*
F×B	AVSD	rs6255362	chr5:116106456-134045067	**4.74**	B	*Ptpn11, Med13l, Tbx3, Tbx5*
F×B	AVSD	rs4217260	chr16:85097188-93538107	2.73	F	
